# Are the Kinetics and Kinematics of the Surf Pop-Up Related to the Anthropometric Characteristics of the Surfer?

**DOI:** 10.3390/s21051783

**Published:** 2021-03-04

**Authors:** Márcio Borgonovo-Santos, Thiago Telles, Jeff Nessler, Marcelo Peduzzi de Castro, Ricardo J. Fernandes, João Paulo Vilas-Boas

**Affiliations:** 1 Centre of Research, Education, Innovation and Intervention in Sport (CIFI2D), Faculty of Sport and Porto Biomechanics Laboratory (LABIOMEP-UP), University of Porto, 4200-450 Porto, Portugal; marcio.santos@riedel.net (M.B.-S.); tellesfef@gmail.com (T.T.); ricfer@fade.up.pt (R.J.F.); 2 Riedel Communications GmbH & Co. KG, R&D Hub Portugal, 4450-718 Porto, Portugal; 3 Laboratory of Aquatic Activities, Physical Education Faculty, Sports Science Department, State University of Campinas, Campinas 13083-521, Brazil; 4 Department of Kinesiology, California State University, San Marcos, CA 92096, USA; jnessler@csusm.edu; 5 LaBClin Neuromusculoskeletal Rehabilitation and Clinical Biomechanics Laboratory, Florianópolis 88015-310, Brazil; marcelocastro@labclin.net.br

**Keywords:** surf, pop-up, GRF, impulse, kinetics, time phases, kinematics, biomechanics

## Abstract

The surf pop-up is a unique and challenging skill, critical to successful surfing. Hypothesizing that anthropometric characteristics of surfers influence the pop-up performance, we aimed to measure kinematics and ground-reaction forces (GRF) during a simulated pop-up motion, and to relate these variables with anthropometric characteristics. Twenty-three male surfers (age: 28.4 ± 10.1 years old; body mass: 68.3 ± 10.8 kg; height: 1.73 ± 0.07 m; time of practice: 12.4 ± 8.9 years; arm-span: 1.75 ± 8.9 m) perform a simulated pop-up in the laboratory, while GRF and 3D motion-capture data were acquired. The duration of the pop-up was 1.20 ± 0.19 s (60% push-up and 40% reaching/landing phase). During the push-up, the hands were placed 0.46 ± 0.05 m apart and generated a relative total peak-force of 0.99 ± 0.10 N/Weight, with symmetrical impulse of 0.30 ± 0.05 N·s/Weight for the dominant and 0.29 ± 0.07 N·s/Weight for the nondominant hand. Elbow angles were not different during the peak force application (110 ± 18° vs. 112 ± 18°, respectively) of the push-up phase. During the landing phase, the feet were placed 0.63 ± 0.10 m apart and generated a relative peak force of 1.63 ± 0.18 N/Weight. The impact force during landing was applied unevenly between the rear foot (28%) and the front foot (72%). In conclusion, most anthropometric-related variables showed no relationship with performance variables, with the exception of an inverse relationship between muscle mass and pop-up total duration. We also observed no differences in upper- and lower-body kinematics between the dominant vs. nondominant hands and among surfers who preferred a regular vs. “goofy-foot” stance. Finally, the force profiles between hands were similar and symmetric, while the lower extremities during the reaching phase were different, with the front foot applying greater force than that of the rear foot.

## 1. Introduction

Wave riding is the essence of surfing, but the successful performance of this highly advanced motor skill cannot be achieved without first completing a series of complex tasks in a dynamic and unstable environment. The “pop–up”, defined as a quick transition from the prone to standing position on a surfboard, is one such task that is critical to surfing performance. While the pop-up motion can be isolated and examined in the laboratory, it occurs in the water as a seamless extension of the paddling motion performed as a surfer catches a wave. While positioning for a wave, several quick and powerful paddling strokes are needed to attain enough speed to allow the wave’s energy to propel the surfer and surfboard forward and down the face of the wave. When the surfer is about to ride the wave, there is a brief but crucial moment during which the surfer must quickly pop-up and begin to perform maneuvers on the wave [1,2,3,4].

The pop-up motion represents a unique challenge to the human motor system, as it must be performed quickly, with sufficient force, on a moving and unstable surface [3]. Adding to this challenge, surfers must learn to successfully perform the pop-up across a wide range of conditions that can change both within and between surf sessions. These conditions include differences in wave size, speed, and shape, often determined by the bathymetry of the break; the period, size, and direction of the swell; and the state of the tide [5]. In addition, the successful surfing performance might be influenced by external factors, including environmental changes such as wind and water temperature [2], as well as internal factors, including strength, fatigue, balance, and thermoregulation [6,7]. Finally, surfers may choose to ride surfboards of differing size, shape, and density, and must therefore adjust their paddling and pop-up behavior accordingly.

The biomechanics of the pop-up motion are unique and complex. This motion is crucial to the surfer’s proper execution of planned maneuvers. The knowledge of surfing pop-up biomechanics might therefore help to inform coaches and athletes seeking to improve performance. The first action of the technique consists of using the upper limbs to push against the surfboard’s deck to propel their center of mass upward relative to the surfboard. Three phases are commonly considered: (i) the push-up phase represents the time between first hand contact with the surfboard up to the point when both hands leave the surfboard; (ii) the transition phase is characterized by the moment when the hands leave the board, and the foot touches the board; and (iii) the reaching phase refers to the foot touching the surfboard up to the surfer achieving stabilization on the surfboard. Male surfers were previously shown to exert an average of 95% of body weight while pushing with their arms during a simulated pop-up in the laboratory [3]. Although there is currently no data to characterize the entire movement, this action is driven by laterality dominance since the surfer must adopt a “regular” or “goofy” posture on the surfboard. Regular surfers place the left foot toward the surfboard nose, while a goofy stance uses the right foot in the front of the surfboard. This stance is most often a half-squatting position with knees flexed. While standing, the rear knee is often stressed in a valgus position due to navigating the accelerating surfboard as it travels down the wave [2].

The complexity of the technique, added to the intervening factors, makes the degree of difficulty for its execution relatively high for surfers of all skill levels. To date, most research in surfing has focused on wave riding and paddling [8,9,10,11,12,13], and the pop-up is often overlooked, despite its recognition as an important aspect of surfing. As surfing has gained worldwide popularity in recent years, there has been a parallel increase in sport-science research attempting to maximize sports performance [14]. Success at any level within surfing requires a high level of skill execution and technical ability [7]. Detailed knowledge of the technique and the physical demands required for proper pop-up are important for preventing injuries, effective coaching, and improving performance. Studies examining full-body motion and both upper- and lower-extremity force during the pop-up are therefore both necessary and lacking.

Previous studies have also identified that certain body characteristics may influence an individual’s performance while surfing [15]. For example, findings related to levels of body-fat mass [16] and height of the center of gravity [17] demonstrate, respectively, an inverse relationship to the ability to paddle and maintain stability. Therefore, anthropometric factors may impact the performance of the pop-up, yet to date, this has not been investigated.

The purpose of this study was to describe kinematics and ground-reaction-force parameters of surfers performing a simulated pop-up movement. In addition, regular and goofy-foot surfers were compared, as were measurements of select anthropometric properties, in order to determine whether any of these factors are associated with the performance of a simulated pop-up movement.

## 2. Materials and Methods

This is an exploratory and descriptive study using anthropometrical measurements, followed by a three-dimensional motion capture, synchronized with ground-reaction forces taken from multiple force platforms in standardized laboratory conditions. The combination of the techniques allowed for a complete, whole-body, biomechanical analysis of the pop-up motion. This study was conducted according to the Helsinki Declaration and approved by the local ethics committee (project number: CEFAD 27.2014). Written informed consent was obtained from all participants involved in the study.

### 2.1. Participants

Twenty-three male surfers (age: 28.4 ± 10.1 years old; body mass: 68.3 ± 10.8 kg; height: 1.73 ± 0.07 m; time of practice: 12.4 ± 8.9 years; arm-span: 1.75 ± 8.9 m) volunteered to take part in this study (Table 1). As inclusion criteria, participants had surfed for the previous two years with a minimum regular practice once per week. Exclusion criteria included any serious musculoskeletal injuries in the last six months. Table 1 shows the participants’ cross-information about hand-dominance and preferred foot base.

### 2.2. Experimental Procedures

Prior to the pop-up analysis, the surfers’ anthropometric measurements and body-composition analysis were obtained, both while in the standing position. Body composition was assessed through multifrequency bioimpedance analysis using the InBody 230 (Biospace Co., Ltd., Seoul, Korea). Together, these tests yielded body mass index (BMI), percentage of skeletal muscle mass (SMM%), percentage of body fat mass (BFM%), and waist-to-hip ratio (WHR).

**Figure 1:**
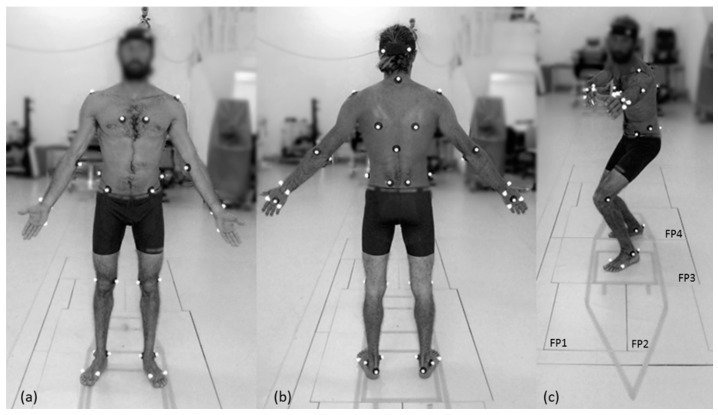
Full-body marker setup: (**a**) anterior view, (**b**) posterior view, and (**c**) force-plate arrangement and surfboard drawing representation.

A 12-camera digital motion-capture system (mocap) (Qualisys, Gothenburg, Sweden) was used to record three-dimensional movement of the participants at a 200 Hz sampling frequency. A volume of approximately 45 m^3^ (5 m long, 3 m wide, and 3 m deep) was calibrated using an L-shaped reference structure and wand according to the manufacturer’s recommendations (0.7 mm standard deviation error calibration mean). Spherical retro-reflective markers were attached to the skin by double-faced adhesive tape, and clusters were fastened with an elastic strap. The full-body model consisted of 46 markers at the following anatomical locations (Figure 1): right anterior head, left anterior head, right posterior head, left posterior head, right acromion, left acromion, right lateral epicondyle, left lateral epicondyle, right medial epicondyle, left medial epicondyle, right styloid process of ulna, left styloid process of ulna, right styloid process of radius, left styloid process of radius, right second metacarpal head, left second metacarpal head, right third metacarpal head, left third metacarpal head, right fifth metacarpal head, left fifth metacarpal head, right scapula inferior angle, left scapula inferior angle, right sternum, left sternum, seventh cervical spinal process, tenth thoracic spinal process, right anterior superior iliac spine, left anterior superior iliac spine, right posterior superior iliac spine; left posterior superior iliac spine, right medium iliac spine, left medium iliac spine, right medial knee (medial epicondyle of the knee), left medial knee (medial epicondyle of the knee), right lateral knee (lateral epicondyle of the knee), left lateral knee (lateral epicondyle of the knee), right lateral malleolus, left lateral malleolus, right medial malleolus, left medial malleolus, right first metatarsal head, left first metatarsal head, right fifth metatarsal head, left fifth metatarsal head, right calcaneus, and left calcaneus.

Ground-reaction-force data were obtained using four force plates (Figure 1c). Two of the force plates, FP1 and FP2, were 60 × 40 cm^2^; while the other two, FP3 and FP4, were 60 × 90 cm^2^ (Bertec, Columbus, OH, USA). The force plates were mounted flush with the laboratory floor, and ground-reaction-force data were acquired at 1000 Hz. The two-dimensional outline of a typical short surfboard (74 inches in length) was created on the laboratory floor using tape. The image was constructed such that each of the four force plates were positioned in a quadrant of the board and could register the force generated by each upper and lower limb in action during the pop-up motion.

**Figure 2:**
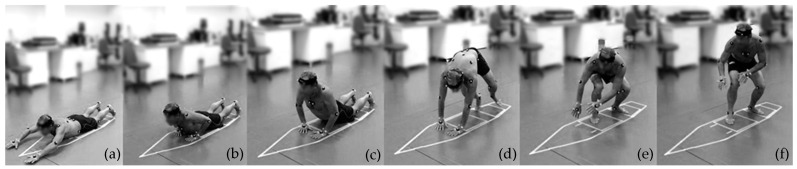
The sequence of movements performed: (**a**) paddle simulation, (**b**) touching hands on the surfboard, (**c**) pushing, (**d**) transition, (**e**) reaching the surfboard, and (**f**) weight stabilization.

The surfers were instructed to familiarize themselves with the representative surfboard and force plates and to adjust their prone position according to their personal preferences. The researcher then explained the procedures for simulating the pop-up movement freely, while respecting the surfboard dimensions on the floor. First, the surfers were asked to simulate 3–4 paddling movements and then performed the pop-up. If the surfer’s hands were not entirely on the force plates we asked the surfer to move fore or aft along the surfboard. This adjustment did not impose any influence on the movement. Surfers were encouraged to direct their gaze forward as if they were surfing. Following this familiarization period, surfers were allowed to perform the entire pop-up motion freely, without any restriction from the researcher (Figure 2).

### 2.3. Data Processing and Analysis

Qualisys Track Manager–QTM version 2.2 (Qualisys, Gothenburg, Sweden) software was used to acquire the 3D kinematic and ground-reaction-force data simultaneously and synchronized. Each reflective track marker was identified using the respective anatomical reference label. The marker reconstruction accuracy reached 100%. After this treatment, the data files were exported using a public-domain binary file format, C3D (Coordinate 3D–C3D.org), which stores 3D data and their associated parameters (i.e., 3D ground reaction forces) in a single file.

Visual 3D Professional version 6.0 (C-motion Inc., Rockville, MD, USA) software was used to process the kinematic and ground-reaction-force data from the C3D files. A full-body biomechanical model was created. All body segments were created based on the markers fixed at the anatomical points. A bilateral marker set and two tracking markers were actually provided for each segment: head, thorax/ab, pelvis, upper-arms, fore-arms, hands, thighs, shank, and feet. The inverse kinematics algorithm was selected to compute the model according to recommendations from the C-motion Visual 3D documentation and applied in each file, taking into account each surfer’s body weight.

The beginning of the pop-up was defined as the point when at least one hand touched the surfboard (FP1 or FP2), right after the simulated sprint paddling. Thereafter, the data-mining process and visual inspection of the motion technique analysis reveal three distinct serial and complementary phases: (i) the push-up phase is defined as the time between first hand contact with the surfboard, followed by the push-up movement, up to the point when both hands leave the surfboard; (ii) the transition phase, which can be performed in two distinct ways: (1) Wipe-transition—the surfer experiences a flight phase (no contact) or immediately after their hands leave the surfboard, one or both of their feet contacts the surfboard—the duration counts the time between hands leave the surfboard until any foot touch the surfboard; (2) Overlap-transition—the surfers’ hands remain in contact with the surfboard while one or both feet contact the surfboard—the duration starts to count when any foot touches the surfboard until both hands leave the surfboard; and (iii) the reaching phase refers to the period between either foot touching the surfboard and the surfer achieving stabilization of their own weight on the surfboard. The stabilization of the weight was defined as the instant when the surfers reached their exact body weight, measured by FP3 and FP4. The reaching phase should result in the surfers’ feet individually positioned front foot on FP3 and back foot on FP4 (Figure 1c and Figure 2f). If this did not happen, the surfer repeated the action, making adjustments until his technique produced the desired motion. The stabilization time defined the end of the pop-up movement.

A specific pipeline script command for Visual 3D was created to identify events that define the pop-up phases, and to extract the related parameters to be analyzed. The principal time-related events and parameters were: (a) hands touch FP1 and FP2; (b) distance between hands using the lateral markers positioned in the metacarpus of the little fingers; (c) pushing-up peak-force result from FP1–2, and the respective elbows angles in this instant; (d) instant when the hands leave FP1–2; (e) feet touched FP3–4; (f) flight phase, the period within the wipe-transition where the surfer has no contact with the surfboard; (g) reaching peak-force resulting from FP3–4 and the respective knee angles; and (h) weight stabilization and the feet base distance (using the lateral markers positioned on the metatarsus of the 5th toes).

The ground-reaction-force values in individual time curves were normalized by individual body mass (N/kg). To calculate the impulse and impact absorption during each phase, the time integral of the force/time curves were calculated.

Data processing generated outputs in text files for statistical analysis. The principal events generated were: hand-touch time, push-peak force (independent and both hands), hand-push-out time, reach time, reach-peak force (independent and both feet), and stabilization. A script was written to automatically extract the events. Events that were not automatically detected were identified manually using force-plate data. We expected the operator variability to be minimal for these cases because the force data were high resolution and events were clear and easy to identify. All the events registered (both manual and automated) were visually inspected to confirm accuracy.

The kinematic data were interpolated using the Piecewise Cubic Hermite Interpolating Polynomial method with 100 samples, to ensure that each trial included the same number of samples. A symmetry function (SF) with temporal dependence was then applied to identify the percent difference between the right X_R_(t) and left X_L_(t) sides, for elbow and knee angle, relative to the respective average range of motion (ROM) [18]. The positive or negative signal indicated the leading side. A score close to zero indicated symmetry (equality) between limbs. Magnitudes of 10% were typically reported in symmetrical populations when other movements were analyzed [18]. Therefore, an asymmetry of 15% or more is thought to indicate substantial asymmetry [18].

SF(t) = (X_R_(t) − X_L_(t))/(0.5 [Range(X_R_(t)) + Range(X_L_(t))]) ∗ 100% (1)

### 2.4. Statistical Analysis

All data were analyzed using the statistical software Statistica 12 (StatSoft©, Tulsa, OK, USA) and Excel 2016 (Microsoft Corp., Redmond, WA, USA) with *p* ≤ 0.05 significance level. All variables were reported with descriptive statistics (mean and standard deviation). For the temporal variables, descriptive statistics were used for analysis, and the pop-up subphases were presented relative to the total duration of the gesture. A significance level (α) of 0.05 was used for the inferential tests. Factorial ANOVA was used to investigate differences in kinematic parameters (elbow and knee angles during the peak-forces) between hand-dominance and stance-feet-base. A one-way ANOVA was conducted to verify the existence of differences between the type of transition and the performance of the pop-up. A dependent-samples *t*-test was used to identify differences between individual hand peak-forces during the push-up phase, and also for the reaching phase comparing front and rear feet peak-force distribution. A multiple linear regression model was applied to analyze the influence of body characteristics (skeletal muscle mass—SMM%; body fat mass—BFM%; body height) on the performance of the pop-up technique (duration). Finally, another multiple linear regression model was used to analyze the influence of the performance of the pop-up technique (duration) on the kinetic variables (ground-reaction-force peak during the pushing and reaching phases).

## 3. Results

### 3.1. Kinematics

**Figure 3:**
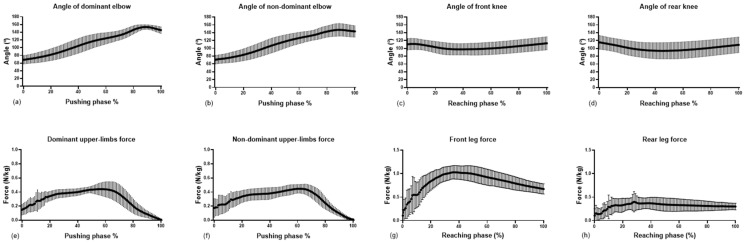
Average and standard deviation of kinematic and force variables during the pop-up. The first line shows (**a**,**b**) as the dominant and nondominant elbows angle during the pushing phase; (**c**,**d**) are, respectively, the front and rear knee angle during the reaching phase. On the second line, (**e**,**f**) show the dominant and nondominant upper-limb ground reaction forces during the pushing phase, while (**g**,**h**) show the front and rear leg ground reaction forces.

At the beginning of the push-up phase, the elbow was flexed at about 70°. From there the elbow progressively extended, and at about 80% of the push-up phase reached maximal extension. Both elbows then exhibited slight flexion up to the end of the push phase (Figure 3a,b). Considering the knee joint, there was a flexion movement of around 15° from the beginning to the middle of the reach phase, followed by an extension movement with similar amplitude until the stabilization (Figure 3c,d). The angular symmetry between dominant and nondominant elbows suggested a similar behavior, SF = 0% ± 7% with a ROM of 18% ± 15%; while between the front and rear knee, SF = 4% ± 12 with a ROM of 20% ± 17%.

Factorial ANOVA showed no interaction effect, F(1,19) = 0.044; *p* = 0.83, or main effects for hand-dominance F(1,21) = 0.121; *p* = 0.73, and the stance-feet-base F(1,21) = 0.633; *p* = 0.435. Further, no differences in elbow angles were detected during the peak-force on the push-up phase between the dominant and nondominant hands (110 ± 18° vs. 112 ± 18°). Finally, no differences in knee angles were observed during the peak-force on the reaching phase between the front and back foot (99 ± 20° vs. 101 ± 14°). In this way, all the kinematic results for dominant and non-dominant limbs, and regular or goofy-stance feet base were reported without distinction for these criteria.

**Figure 4:**
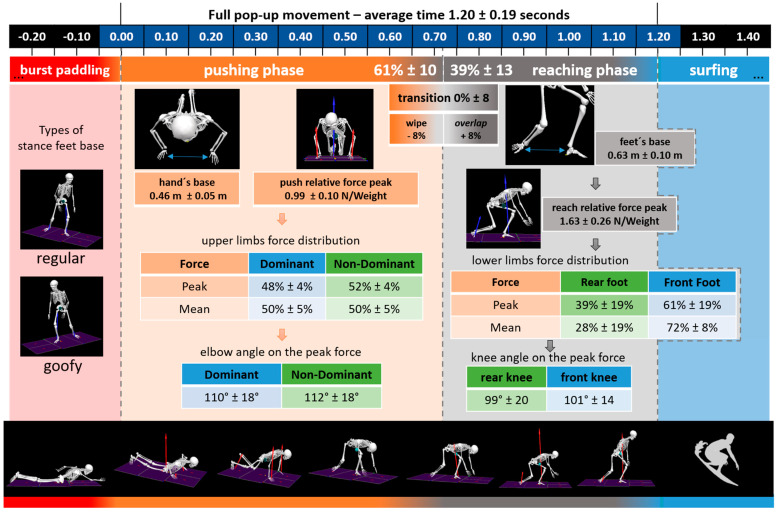
Infographic of pop-up movement results.

The entire pop-up movement (Figure 4) showed an average duration of 1.20 ± 0.19 s. Deconstructing the technique into elementary parts, 61 ± 10% (0.71 ± 0.08 s) of the time was spent in the push-up phase; 0 ± 8% (0 ± 0.09 s) in the transition phase; and 39 ± 13% (0.48 ± 0.22 s) in the reaching phase. During the transition phase, 13 out of the 23 surfers used the wipe-transition (with a flight phase of 0.05 s ± 0.03 s), while 10 surfers used the overlap transition. No differences were found, F(1,21) = 0.02; *p* = 0.87, related to the total pop-up execution time between these two variations in technique.

### 3.2. Ground Reaction Force

During the push-up phase, the hands-base width was 0.46 ± 0.05 m and achieved a relative total peak-force of 0.99 ± 0.10 N/kg. The ground reaction forces increased up to about 60% of the phase and then decreased to the end of the motion (Figure 4). Factorial ANOVA showed that the nondominant hand exerted significantly greater peak-forces during the push-up phase, on average, than the dominant hand (52 ± 4% vs. 48 ± 4%, respectively, t(22) = −2.27; *p* = 0.03). However, the entire force/time curve was equally distributed for both hands (50% ± 5% each), with similar impulses, t(22) = −1.36; *p* = 0.18, of 0.30 ± 0.05 N·s/kg, for the dominant hand, and 0.29 ± 0.07 N·s/kg for the nondominant hand.

During the reaching phase, the surfers positioned their feet 0.63 ± 0.10 m apart, and generated a peak 1.63 ± 18 N/kg of impact force. The pattern of force progression was very distinct between the rear and front limbs (Figure 4). Factorial ANOVA showed significant differences in the landing peak-force distribution between feet, t(22) = −2.76; *p* = 0.01; where 39 ± 19% occurred in the rear foot and 61% ± 19% occurred in the front foot. The entire force/time curve distribution reinforced this difference, t(22) = 13.09; *p* < 0.01, where 28 ± 8% occurred in the rear foot and 72% ± 8% occurred in the front foot, with the respective impact absorption of 0.32 ± 0.11 N·s/kg, and 0.81 ± 0.13 N·s/kg.

The multiple regression model indicated no association between the performance of the pop-up and the ground-reaction-force variables (r = 0.18, *p* = 0.71).

### 3.3. Anthropometry

On average, participants demonstrated body composition values as follows: BMI 23 ± 3; SMM% 49 ± 3%; BFM% 14 ± 5%, and waist-to-hip ratio 0.84 ± 0.05. An inverse relationship between SMM% and the transition phase time was found, r = −0.50; *p* = 0.01. A similar relationship was found for feet-base and the rear-knee angle r = −0.50; *p* = 0.01. No correlations were found for pop-up performance versus body characteristics: SMM% (r = 0.15; *p* = 0.47), BFM% (r = 0.05; *p* = 0.81), and height (r = 0.09; *p* = 0.67). No significant regression weights were found after controlling for each variable, R^2^ = 0.15; F (3,19) = 1.12; *p* < 0.36.

## 4. Discussion

The purpose of this study was to analyze the kinematics and kinetics of the pop-up movement during simulated conditions, using a specially designed configuration of force plates representing a typical surfboard. This configuration allowed for decoupling all the ground reaction forces into an individual analysis of each body limb’s contribution, associated with 3D kinematic information from the mocap system. These data were then used to characterize critical aspects of the pop-up technique from a full-body-model biomechanical perspective.

There were four primary results from this study. First, no differences in upper and lower body kinematics were observed between the dominant vs. nondominant hands, or among surfers who preferred a regular vs. goofy-foot stance. Second, ground reaction peak-forces generated by the hands during the push-up phase were different and greater for the nondominant hand. However, while analyzing the impulse over the entire duration of the phase, the force profiles between hands were very similar and symmetric. Third, ground reaction forces generated by the lower extremities during the reaching phase were significantly different, with the front foot applying greater force than that of the rear foot. Finally, a significant inverse relationship was found between skeletal muscle mass percentage and total duration of the pop-up (r = −0.50; *p* = 0.01), but no other relationships were found between anthropometric variables and performance. Additional results included the observation of two distinct pop-up techniques utilized by participants, defined by either the presence (57% of participants) or absence (43% of participants) of a brief flight phase.

### 4.1. Push-Up Phase

The pop-up technique is very fast and involves a coordinated sequence of movements that start with strength and the powerful push-up phase [1]. According to the current data, the surfers pushed, using the hands alone, with a total force equal to their full-body weight. Though a relatively small difference was detected in peak-force between the two hands, the full time-force curve for the push-up phase was very similar. A similar force distribution between hands may contribute to improved balance for the pop-up execution, preventing unwanted instability of the surfboard on the water surface. The current results demonstrate that the relative push peak-force was slightly higher than the values reported in the literature [3], 0.95 N/Weight for men and 0.81 N/Weight for women. The inclusion of impulse values appears to be helpful in understanding the work involved in this explosive task.

### 4.2. Transition Phase

Surfers in this study used one of two distinct techniques during the transition phase, as they shifted their body from the horizontal to the vertical standing position. These observed transition types included the wipe and overlap techniques (Figure 4). The data indicated that using either technique did not result in any differences in the velocity of the entire pop-up motion. Conversely, an inverse correlation was found between SMM% and the transition-phase duration. This suggests that surfers with higher muscular mass can perform the transition phase faster, and may have greater control of the type of transition used. However, when one considers the dynamic and unstable environment in which the pop-up is executed, it may be advantageous for the surfer to maintain contact with the board at all times.

### 4.3. Reaching Phase

When surfers placed their feet on the simulated surfboard, the maximal load reached approximately 160% of their body weight. While this value may appear high, it is important to note that this was recorded in a laboratory environment; these values may be different in the water, depending on the type of board and oceanic conditions. During the landing phase of the simulated pop-up, the distribution of the load, unlike the push-up phase, was quite uneven. This difference may be a consequence of the unstable conditions experienced by the surfer in water. In particular, applying a greater relative force with the front foot will help to reduce the pitch angle of the board and keep it flat against the surface of the water. This action would help to increase the drop velocity and keep the surfer’s body perpendicular to the surfboard, possibly leading to more balance and control of the surfboard through their feet. It may also help to propel the surfer’s center of mass down the slope of the wave.

On the other hand, more weight on the rear foot would serve to increase the pitch angle, thereby increasing the drag resistance, and slowing down the surfboard’s velocity. It is interesting to note that most participants applied a greater percentage of body weight on their front foot (about 60%). This suggests that fore–aft distribution of weight during the reaching phase may be an important factor in increasing the drop velocity while riding a wave.

The stance of a surfer is described as a half-squat position with knees flexed 30–80°, with the rear knee in a valgus position [2]. The current results generally support this description, but overall showed higher knee-flexion angles and a lack of consistency among surfers regarding foot placement. Also, foot placement did not appear to be related to any variable that was analyzed here. It is speculated that the feet base length chosen by the surfers could be related to the surfboard size and/or the surfer’s comfort in the standing position. In this position, the rear and front knee angles showed similar values in magnitude, but indeed, the legs were positioned differently. Accordingly, the rear leg showed slight internal rotation, a pronated foot, and the knee pointing to the middle of the surfboard, while the front leg held a normal squat position. With this description, it is easier to understand the observed relevant correlation between the feet base and the rear knee angle. The closer the feet are, the lower the knee angle should be, in order to compensate for the shorter distance.

### 4.4. Limitations

This work characterized a simulated pop-up movement performed by surfers as they shifted from the horizontal to the standing position on the laboratory floor, which may be somewhat different from performing a similar motion in the ocean. In particular, the breaking wave generates greater challenges, and the relevance of these ecological differences cannot be estimated. In addition, the simulated test utilized a stationary paradigm, which is different from the dynamic and changeable conditions created by the waves. However, limitations in equipment have precluded testing in the ocean, and the analyses described here were performed under highly controlled and repeatable conditions, which are very difficult to achieve in the ocean. Further, we recommend caution on interpreting our GRF results, as the surfers might change their pop-up technique depending on their need to accelerate or brake the surfboard. We opted to let the participants choose their pop-up technique because, in a real surfing environment, they also choose their strategy freely. Additional instruction, such as to recommend accelerating or braking the board, would decrease the ecological validity of our experimental protocol. We believe this condition would mimic the most common pop-up style performed by the surfer. In the present study, we recorded three pop-up trials and arbitrarily extracted one for analyses. Although we did not explore the within-subject reliability, our visual analysis of the trials suggests intrasubject consistency for the movement.

## 5. Conclusions

A simulated pop-up motion performed by surfers in the laboratory was analyzed using motion capture and specially configured force platforms. The pop-up was characterized by three sequential phases: push-up, transition, and reaching. During the push-up phase, the upper limbs acted symmetrically and generated forces equivalent to the full-body weight to initiate the change from a prone to a standing position. During the transition phase, approximately 57% of participants exhibited a brief flight phase, whereas 43% maintained contact with the simulated board throughout the entire motion. During the reaching phase, the front lower limb applied greater force to the board, presumably maintaining a lower pitch angle and reducing drag force as the surfer accelerated down the wave and began to maneuver. These data provide biomechanical insight into this motion analysis. They may apply to the training and coaching of surfing athletes to improve their surfing performance by focusing on their pop-up technique.

## Figures and Tables

**Table 1:** Cross-information among the surfers’ hand dominance and stance feet base.

N = 23	Stance Feet Base		
Hand Dominance	Regular	Goofy	Total
Right	10	7	17
Left	2	4	6
Total	12	11	23

## Data Availability

https://www.youtube.com/user/GigantFire (accessed on 23 February 2021).
